# Comparative Study of Nest Architecture and Colony Structure of the Fungus-Growing Ants, *Mycocepurus goeldii* and *M. smithii*

**DOI:** 10.1673/031.007.4001

**Published:** 2007-06-25

**Authors:** C. Rabeling, M. Verhaagh, W. Engels

**Affiliations:** ^1^ Zoologisches Institut, Universität Tübingen, Auf der Morgenstelle 28, D-72076 Tübingen, Germany; ^2^ Staatliches Museum für Naturkunde, Erbprinzenstr. 13, D-76133 Karlsruhe, Germany; ^3^ Current address: Section of Integrative Biology, University of Texas, 1 University Station C0930, Austin, Texas, U.S.A. 78712-0253

**Keywords:** natural history, nest excavation, soil dwelling arthropods, soil ecology

## Abstract

Nest architecture and demography of the non leaf-cutting fungus-growing ant species *Mycocepurus goeldii* and *M. smithii* (Attini: Formicidae) were studied in an agroforest habitat near Manaus, Brazil during the excavation of 13 nests. Both species built their nests in two different ways. The first type possessed a “tree-like” architecture, in which a vertical tunnel led downwards and lateral tunnels branched off at 90° angles from the main tunnel, with a chamber at the end of each side branch. Alternatively, other nests displayed a “necklace-like” architecture, where the main tunnel also led down vertically, but entered each chamber from the top and exited it at the bottom, resulting in an architecture where chambers appeared like pearls on a necklace. The nest systems of *M. goeldii* and *M. smithii* consisted of 1–21 or 1–15 chambers, respectively. Of 199 excavated chambers, 57 % contained fungus-gardens. Chambers not containing fungus gardens were filled with organic matter from decaying fungus gardens or earthworm feces. Only *M. smithii* workers deposited loose soil in abandoned chambers during the construction of new nest chambers. Workers of *M. smithii* constructed significantly smaller chambers than those of *M. goeldii.* In both species, fungus garden-containing chambers were larger than non-garden chambers and were homogenously distributed in the soil between 17 cm and 105 cm depth. Neither fungus gardens nor abandoned chambers were encountered more frequently in deeper or shallower soil strata indicating that ants of both species did not abandon shallower versus deeper chambers, or move the colony to deeper soil layers with increasing colony age. Fungus gardens were suspended from the ceiling of the subterranean chambers and originated as small mycelial tufts. Through continual addition of organic debris, the tufts first grew vertically to strands before they expanded laterally until most of the chamber volume was filled with fungus garden curtains. New garden chambers were found at depths ranging from 17 to 83 cm, suggesting that new garden chambers were not constructed in deeper soil strata with increasing colony age. The size of *M. goeldii* and *M. smithii* colonies was dependent on their age. Worker counts varied between a few individuals in recently founded colonies and 1352 workers in an adult *M. goeldii* colony. The ratio of worker number per fungus garden chamber was higher in *M. goeldii* colonies than *M. smithii* colonies; the *M. goeldii* colonies were more populous. Both species were oligogynous with a maximum of four and three queens observed in a single chamber of *M. goeldii* and *M. smithii,* respectively. The reproductive status of each queen was unknown. In both species the ratio of brood to workers was approximately 2:3. Larvae and pupae were unequally distributed throughout the nest, but were only located in chambers containing a garden. Their location in the chamber was dependent upon the developmental state of the fungus garden.

## Introduction

**Figure 1.**
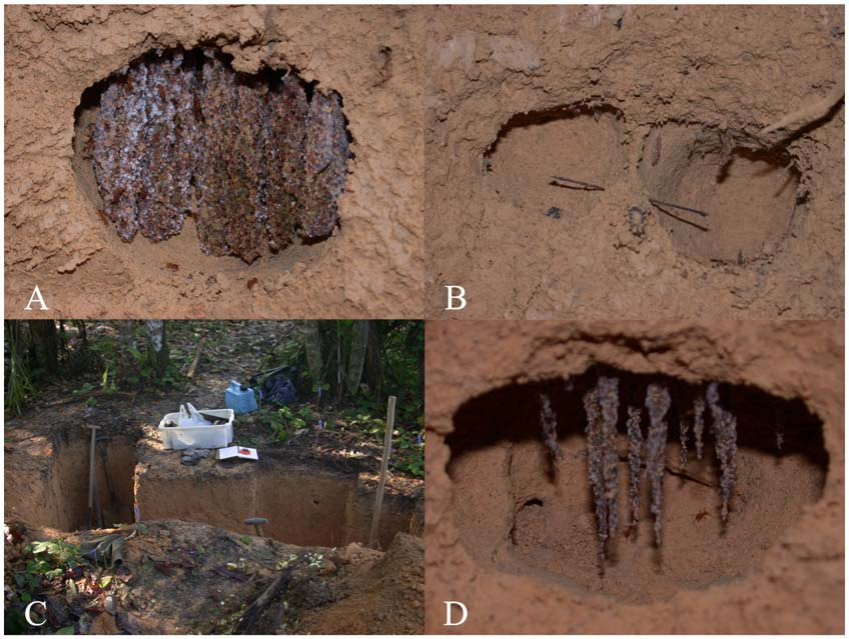
A-D: Overview and details of a *Mycocepurus goeldii* nest excavation. (A) Mature fungus garden in a nest chamber. (B) Adjacent chambers with fungus garden removed; a connecting tunnel between them is marked with a stick. (C) Excavation of two adjacent nests: on the far left side the excavation of a nest system is complete, on the right side it has just begun. (D) Mycelial strands of an incipient garden hang from the ceiling of a recently constructed chamber. Photos by C. Rabeling.

The symbiotic relationship between ants and basidiomycete fungi is the unique behavioral character of the approximately 210 extant fungus-growing ant species of the monophyletic, New World tribe Attini (Schultz and Meier 1995; Brandão and Mayhé-Nunes 2001). Based on their foraging behavior, most Attini species belong to either of two ecological guilds; the herbivorous leafcutter ants (*Atta* and *Acromyrmex*) grow their fungus gardens (Figure 1a) on fresh leaf substrate, whereas the non-leafcutting species collect organic debris from the leaf litter stratum to nourish their fungal cultivars (Weber 1972).

To understand the origin of the fungus growing habit, the more plesiotypic attine lineages are expected to be most informative (Mueller 2002). The genus *Mycocepurus* is among the most plesiotypic genera (Schultz and Meier 1995), but detailed natural history observations have been lacking because their nests are difficult to excavate due to their small nest chambers, which –are hidden in deeper soil strata (but see Fernández-Marín et al. 2005).

The two focal species of this study, *M. goeldii* and *M. smithii*, occur in remarkably high nest densities in secondary habitats in the Brazilian Amazon. We hypothesized that these debris-collecting gardening ants might play an important ecological role in soil physics and chemistry of Amazonian agroforestry systems (Rabeling et al. 2004). To investigate their ecological impact and contribute additional data towards the quest for the origin of fungus cultivation in attine ants, basic ecological and natural history data were collected in a quantitative, standardized fashion. This report is the first part of our study: a detailed, comparative survey of the nest architecture and colony structure of *M. goeldii* and *M. smithii* through nest excavations. Data from standardized excavations provide the first comparative insights into the nearly unknown biology of the genus *Mycocepurus* in the Brazilian Amazon. We hope that our study will stimulate the use of standardized excavation methods to explore the ecology and natural history of soil dwelling ants.

## Materials and Methods

Fieldwork was conducted from February to July 2003 at two experimental agricultural field sites of the Embrapa-Amazônia Ocidental (Empresa Brasileira de Pesquisa Agropecuária) near Manaus, Amazonas, Brazil. Nest excavations were made at the headquarters, north of the Rio Negro, located at kilometer 28 of Highway AM 010 (2°53′S 59°59′W; 40–50 m asl) and at the experimental site south of the Rio Negro, named Embrapa-Caldeirão (3°15′S 60°14′W; 30 m asl). The field site at the Embrapa headquarters can best be described as an agroforestry system and is characterized by an assemblage of different fruit, nut and palm trees, including the Brazil-nut tree (*Bertholletia excelsa),* rubber tree (*Hevea brasiliensis*), coconut palm (Cocos *nucifera*), urucum (*Bixa orellana*), peach palm (*Bactris gasipaes*), cupuaçu (*Theobroma grandiflorum*) and several citrus species (e.g. *Citrus* *limon*, *C. sinensis*). The understory was composed of kudzu (*Pueraria phaseoloides*, Fabaceae), a species of Poaceae and three species of Melastomataceae. At Embrapa-Caldeirão, *M. goeldii* colonies were encountered in a manioc (*Manihot esculenta*) plantation, whereas *M. smithii* colonies were found at the edge of a secondary forest between herbaceous growths.

Local rainfall variability is high in central Amazonia (Ribeiro and Adis 1984). Average annual rainfall at the Embrapa headquarters site was 2554 mm (± 273) between 1971 and 2000 with higher rainfall in the second half of the period (1971–1985: 2408 ± 188 mm, 1986–2000: 2700 ± 270 mm). In the nearby city of Manaus, average annual rainfall over the same period was 2313 mm (1971–1985: 2234 ± 219 mm, 1986–2000: 2392 ± 344) (Martius et al. 2004). Normally in this region not more than 1–2 months per year have rainfall below 100 mm (Ribeiro and Adis 1984) but during four months in 1997, rainfall remained below this threshold (Martius et al. 2004). Thus, the climate of the study region can be designated as humid to perhumid. Average monthly temperatures varied between 25 and >28°C, with a 30-year average of 26.0°C (Hanagarth et al. 2004).

Soils in both sites are characterized as “Latossolo amarelo álico”, a type of xanthic ferralsols (sensu FAO/UNESCO, 1990) with a very clayey texture. These soils are common in the Central Amazon and especially in this agricultural district (Distrito Agropecuário da SUFRAMA; sensu Rodrigues et al. 1971, 1972). The pH-values of the topsoil layer vary between 3.7 and 4.2 (measured in CaCl2) and carbon-contents range from 2.0 to 4.5 % (Martius et al. 2004; Martius unpublished data).

Mature *Mycocepurus* nests can be easily recognized by their nest mounds made of bright clay from deeper soil layers, which contrast with the darker soil and leaf litter on the surface. Small nests were located by removing the lower vegetation and carefully cleaning the leaf litter from the soil.

Preliminary nest excavations were conducted under the expectation that *Mycocepurus* colonies only have one to a few nest chambers, as described in various publications (Wheeler 1907; Luederwaldt 1918, 1926; Eidmann 1936; Weber 1945; reviewed by Kempf 1963). However, inhabited chambers were frequently found in close proximity to one another, sometimes only a few cm apart and in some cases adjacent chambers were connected by tunnels (see results; Figure 1b). Additionally, several chambers did not contain a gyne and some chambers that did contain a gyne were encountered at a depth of nearly one meter, which made it unlikely that these chambers were dug by a founding queen.

These observations made it necessary to invest in more extensive excavation methods. Preliminary results revealed that a volume of one cubic meter was a suitable size for *Mycocepurus* nests (Figure 1c). Consequently, in all subsequent excavations (except see below), the following standardized protocol was used. A trench of one-meter depth was dug at a distance of 50 cm from the nest entrance. Thin vertical slivers of soil were sliced away with a sharpened spade in the direction of the nest entrance until the first chamber was encountered. The digging was stopped 50 cm behind the nest entrance or when no additional chambers of the same nest could be found. Test excavations at our field sites near Manaus showed that nest chambers were only exceptionally encountered in depths greater than one meter and therefore, the standardized excavations were limited to a depth of one meter.

All *Mycocepurus* specimens were collected in 95% ethanol and their fungal gardens were oven-dried at 40°C. Only nest chambers with ants and/or fungus garden were included in the colony size counts of nest systems to ensure that the chambers could be positively identified as belonging to a *Mycocepurus* nest. Ants and brood from partially destroyed chambers were not included in demography counts because we could not be sure that representative numbers of individuals were collected. This accounts for the difference in the number of garden chambers used in the comparison of colony demography and chamber measurements between *M. goeldii* and *M. smithii* in Table 2.

**Table 1.**
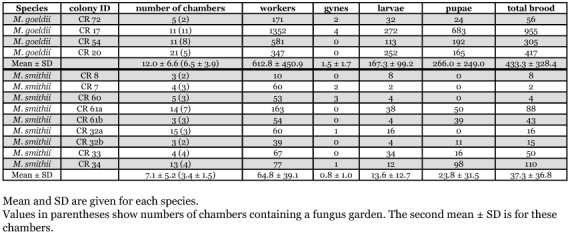
Interspecific comparison of chamber and individual numbers in single nest systems.

In order to investigate the nest architecture the volume of 199 nest chambers, their position in the soil and their depth from the chamber ceiling to the ground were measured. Measurements were only taken if the chamber shape was not destroyed during the digging process. Further data on nest chamber measurement (n = 87) and average number of ants per chamber were collected by additional diggings, which did not follow the described protocol, and added to the results of the standardized excavations (n = 112).

**Table 2.**
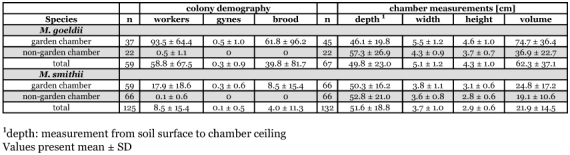
Interspecific comparison of colony demography and chamber size of all chambers collected.

These additional data are from single-chamber nests or chambers, which could not be assigned to a nest system.

## Results

### Nest mounds

**Figure 2.**
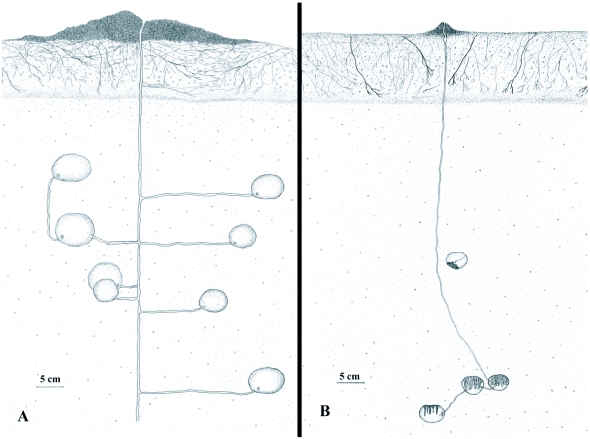
Two nest architectures found in *Mycocepurus* nests. (A) Tree-like architecture of a *M. goeldii* nest (CR 17). Eight of the eleven total fungus chambers are depicted in the cross section; the fungus gardens are not shown. (B) Necklace architecture of a *M smithii* nest (CR 7). The nest consisted of four chambers: the three lowermost ones were filled with a fungus garden, while the deepest chamber contained only a few mycelium strands, characteristic for recently built chambers. The uppermost chamber was abandoned, filled with organic matter from the decaying fungus garden and first rootlets started to grow into the cavity. Because workers fill exiting tunnels of chambers with exhausted and/or parasitized fungus gardens with soil, the chamber was no longer connected to the main tunnel. Both cross sections are drawn to the same scale. Drawings by S. C. Cappellari.

*Mycocepurus* nest mounds consisted of fine grains of soil and had an irregular shape. Frequently, small pseudo-chambers of approximately 0.5 cm diameter were found at the base of the mound (Figure 2b), which were used for storage of particles collected during foraging. The entrance was a small hole of 2–3 mm diameter located in the center or at the periphery of the mound. The amount of soil brought to the surface appeared to be larger in *M. goeldii*, however, the average dry weight ±SD was not significantly different between the two species (for *M. goeldii* 160.1 ± 183.2 g, n = 16; for *M. smithii* 7.3±4.6 g, n = 5; two-tailed t-test on average values: t = 1.774, d.f. = 19, p = 0.092). In *M. smithii* nests abandoned fungus chambers were frequently filled with loose soil. This was never observed in *M. goeldii* nests.

### Nest architecture

Four *M. goeldii* nest systems and nine *M. smithii* nest systems were carefully excavated, revealing two different types of nest architecture used by both species. The “tree-like” architecture (Figure 2a) was characterized by a vertical main tunnel and lateral tunnels which branched off at 90° angles, with a chamber at the end of each side tunnel. Nests with a “necklace-like” architecture (Figure 2b) also had a main tunnel, leading vertically downwards, but the chambers were lined up along the main tunnel, which entered at the top and exited at the bottom of each chamber.

A total of 112 *Mycocepurus*-chambers (Table 1) were excavated by standardized diggings. The number of chambers varied from 1–15 for *M. smithii* and 1–21 for *M. goeldii*, depending on the colonies' development. Using data from additional, non-standardized excavations, a total of 199 nest chambers of *M. goeldii* (n = 67) and *M. smithii* (n = 132) were excavated, of which 111 contained fungus and 88 did not (Table 2).

Of the 88 non-fungus chambers, 33 (38 %) were filled with a mixture of organic substance resulting from the decaying fungus garden and soil and 29 (33 %) contained earthworm feces. In 13 (15 %) chambers, *M. smithii* workers deposited loose soil apparently excavated during construction of new chambers, a behavior that was not observed in *M. goeldii* nests. Twelve (14 %) non-fungus chambers were completely empty and a single *M. goeldii* chamber was found to contain caterpillar feces, which serve as important fungus substrate in this species (Rabeling 2004).

### Mycocepurus goeldii

The average number of chambers per *M. goeldii* nest system, including garden and non-garden chambers, was 12 about half of which contained fungus chambers (Table 1). In three of the four nest systems excavated 83% of the chambers contained fungus gardens. The fourth nest system (nest CR 17, Figure 2a) had fungus gardens in all of its 11 chambers. The deepest and shallowest chambers were found at 83 cm and 21 cm depth, respectively.

During the excavation of nest CR 17, the 2–3 mm wide vertical main tunnel was followed continuously for a distance of 25 cm. Along the main tunnel, four horizontal side tunnels were encountered, each of which led to a single fungus chamber. A fifth tunnel connected one of these chambers to an additional chamber, which was not directly connected to the main tunnel (Figure 2a). All chambers were located at a distance of at most 14 cm from the main vertical tunnel.

### Mycocepurus smithii

Nine nest systems were excavated, revealing a total of 64 chambers, of which 31 (48%) contained fungus garden. The average number of chambers per nest system was 7.1 and the average number of garden chambers was 3.4 (Table 1).

The excavation of nest CR 7 revealed four chambers, three of them with a fungus garden (Figure 2b). The lowermost chamber contained only few mycelium strands, which is characteristic of recently built garden chambers (Figure 1d, 2b). The uppermost chamber was abandoned and contained the organic mass of a decaying fungus garden and some rootlets growing into the cavity (Figure 2b). Because, *Mycocepurus* workers plug tunnels coming from chambers with decaying or parasitized fungus garden with soil, the chamber was no longer connected to the main tunnel.

### Species comparisons

The total numbers of garden versus non-garden chambers per nest system was not significantly different between species (one-tailed t-test on average values: t = 1.44, d.f. = 11, p = 0.089). The nine *M. smithii* nest systems had significantly fewer fungus chambers per nest system than the four *M. goeldii* nests (one-tailed t-test on average values: t = 2.121, d.f. = 11, p < 0.05).

### Chamber Measurements

#### Mycocepurus goeldii

A total of 67 chambers in *M. goeldii* nests were measured, of which 45 (67 %) contained a fungus garden. In an excavation, chambers without a garden were found to occasionally contain a single ant. These chambers were therefore assumed to be connected to the nest system and were considered to be a part of it.

Fungus chambers in *M. goeldii* nests had a significantly higher volume than non-fungus chambers (Table 2) (two-tailed t-test on average values: t = 4.396, d.f. = 65, p < 0.0001).

The average depth of garden chambers and non-garden chambers is shown in Table 2. The average depth of garden and non-garden chambers was not significantly different (two-tailed t-test on average values: t = -1.887, d.f. = 65, p = 0.064). This suggests that *M. goeldii* do not move the nest into deeper soil strata with increasing colony age, nor preferably abandon deeper or shallower chambers.

### Mycocepurus smithii

A total of 132 chambers were excavated, with exactly half of them (n = 66) containing a fungus garden (Table 2). The average volume of chambers without a fungus garden significantly smaller than chambers with a garden (two-tailed t-test on average values: t = 2.261, d.f. = 128, p < 0.05).

The mean depth of garden chambers and non-garden chambers is shown in Table 2. The average depths of fungus versus non-fungus chambers in *M. smithii* nests did not differ significantly (two-tailed t-test on average values: t = -0.76, d.f. = 130, p = 0.449), indicating that *M. smithii* also does not preferably abandon shallower versus deeper chambers during the colony's lifecycle.

### Species comparisons

In both species, the volume of chambers containing fungus was greater than the volume of non-fungus chambers. Both garden and non-garden chambers were significantly smaller in *M. smithii* than those of *M. goeldii* (two-tailed t-test on average values for garden chambers: t = 9.468, d.f. = 107, p < 0.0001; two-tailed t-test on average values for non-garden chambers: t = 4.906, d.f. = 86, p < 0.0001).

The volume of fungus chambers was three times larger in *M. goeldii* than in *M. smithii* (Table 2) which is not surprising, because *M. goeldii* colonies contained significantly more individuals on average than *M. smithii* (Tables 1 & 2). Additionally, *M. goeldii* workers are significantly larger (average mesosoma length 2.659 ± 0.096 mm) than those of *M. smithii* (average mesosoma length 2.315 ± 0.094 mm; two-tailed t-test on average values: t = -5.099, d.f. = 17, p < 0.0001) and heavier (dry weight *M. goeldii* 0.203 ± 0.012 mg; *M. smithii* 0.119 ± 0.005 mg; two-tailed t-test on average values: t = -20.102, d.f. = 17, p < 0.0001; Verhaagh and Schulz unpublished data for n = 10 workers for each species). The volume of *M. smithii* fungus chambers were similar to those reported from Puerto Rico (range from 3.5 cm^2^ to 38.0 cm^2^; Fernández-Marín et al. 2005).

*M. goeldii* and *M. smithii* colonies built their fungus and non-fungus chambers at similar soil depths (two-tailed t-test on average values for garden chamber depths: t = -1.209, d.f. = 109, p = 0.229; two-tailed t-test on average values for non-garden chamber depths: t = 0.794, d.f. = 86, p = 0.43).

### Fungus garden organization and development

In both *Mycocepurus* species studied, the fungus gardens were suspended from the chamber ceiling (Figures 1a, 1d) and not from roots, as it is common in other attines. The garden never touched the chamber walls. The brood was located within small mycelial pockets. In 8 of 111 garden chambers the ants had apparently only recently began to cultivate a fungus garden, as only a single mycelium strand was suspended from the chamber ceiling (Figure 1d) and a single ant was observed to cultivate it using organic debris.

### Colony size

The number of individuals per colony depended on the number of chambers per nest and therefore on colony age. Our data represent a minimum estimate for colony sizes, because some workers and gynes escaped into tunnels when a chamber was opened.

The number of adult workers and brood were unequally distributed between nest chambers depending on the development of the fungus garden in the chambers. In recently constructed chambers, which did not contain a mature fungus garden, as well as in chambers with only a few mycelium strands hanging from the ceiling, only a few workers, no brood and no gyne were present. New chambers were found in different depths ranging from 17 cm to 83 cm. Consequently, chamber depth in the soil seemed not to correlate with the time since construction in a colony lifecycle.

### Mycocepurus goeldii

The total population found in the four excavated nests varied between 171 and 1352 workers (Table 1). The total and average numbers of workers, gynes, larvae and pupae found in *M. goeldii* nests are shown in Table 1. Table 2 shows the average number of workers, larvae and pupae per garden chamber. The highest number of workers in a colony was encountered in colony CR 17, in which we found 1352 workers, 4 gynes, 272 larvae and 683 pupae (Table 1). About 90 % of the brood (larvae as pupae) was found in only four chambers and three chambers did not contain any brood at all. The four gynes were distributed over three chambers and the number of workers ranged from 12–202 per chamber. In oligogynous colonies several gynes could be observed in the same nest chamber. The highest number of gynes in a single chamber was four. Whether all of these gynes were reproductively active was not determined.

*M. goeldii* chambers filled with a large fungus garden had significantly more workers than those containing smaller, presumably more recently founded gardens (two-tailed t-test on average values: t = 2.497, d.f. = 35, p < 0.05), but in general the number of individuals per nest chamber varied considerably (Table 2).

### Mycocepurus smithii

The total and average numbers of workers, gynes, larvae and pupae found in *M. smithii* nests are shown in Table 1. The average number of workers and brood per garden chamber is shown in Table 2. The largest *M. smithii* colony (CR 61) had a total of 163 workers, 38 larvae and 50 pupae located in 7 garden chambers (Table 1). The gyne(s) of this nest escaped during the excavation. Other colonies were found to be oligogynous with up to 3 gynes per nest (CR 60) and a maximum of 2 gynes in a single chamber. *M. smithii* worker counts were not found to be significantly higher in garden-filled versus recently constructed nest chambers (two-tailed t-test on average values: t = 1.569, d.f. = 57, P = 0.122).

### Species comparisons

*M. goeldii* colonies were more populous than *M. smithii* colonies. The average number of workers and brood were both significantly greater in *M. goeldii* chambers (two-tailed t-test on average values for worker number: t = 8.491, d.f. = 94, p < 0.0001; two-tailed t-test on average values for brood number: t = 4.182, d.f. = 95, p < 0.0001).

## Discussion

This study provides the first comparative data on nest architecture and colony size of two sympatric *Mycocepurus* species. The comparative approach used systematic methods that contribute to a better understanding of the natural history of these poorly studied, debris collecting species of fungus-growing ants and will help to quantify the influence of non-leafcutting Attini on nutrient cycling processes. Some attine species cannot be classified as either debris collectors or herbivores, but are found at intermediate positions along this trophic continuum. Primarily debris collecting species were occasionally observed to cut flower petals (Wheeler 1907; Kempf 1963), collect seeds (Weber 1945, 1946; Pizo and Oliveira 1999, 2000; Kaspari 1993; Feldmann et al. 2000; Leal and Oliveira 2000), fruit pulp (Oliveira et al. 1995) or even fresh leaves (Weber 1972; Mayhé-Nunes 1995; Feldmann et al. 2000). But ultimately, the different foraging items serve as substrate for the fungal symbiont, are broken down and the nutrients are returned to the surrounding soil, where they can be used by other decomposers or primary consumers. It is likely that the debris collecting attines have a beneficial effect on soil ecosystems, without being agricultural pests.

### Nest mounds

The external appearance of attine nests is an important character, which differs between genera and, in some cases, enables identification to the genus level (Weber 1982). In *Mycocepurus*, the mounds are of irregular shape, depending largely on whether rainfall has washed away parts of the soil. Irregularly shaped mounds can also be found in other attines, such as *Myrmicocrypta* (Weber 1972). Unique mound characteristics, like turrets of organic matter (e.g. *Trachymyrmex relictus*) or circular craters (*Mycetophylax emeryi*) have not been observed in *Mycocepurus.* Therefore it is not possible to identify *Mycocepurus* nests based solely on the shape of the mound.

The observed mound weights of *M. goeldii* and *M. smithii* nests did not adequately reflect the different volumes of each species' nests. While a significant difference in mound weight was not found, it appears that these two species have different nest construction behaviors. *M. smithii* seems to store part of the excavated soil in abandoned fungus chambers resulting in disproportionately smaller mounds while *M. goeldii* workers seem to bring excavated soil of new chambers nearly completely to the surface, resulting in much larger nest mounds than in *M. smithii.*

### Number of nest entrances

*M. smithii* nests had one entrance with a single tunnel leading from the entrance to one or several nest chambers, which were either lined up directly along the main tunnel or connected to the main tunnel via lateral tunnels. This observation is consistent with those made by Fernández-Marín et al. (2005) in Puerto Rican *M. smithii* colonies.

However, high numbers of nest entrances in *M. smithii* populations have previously led to contradicting reports about the number of entrances per nest in *M. smithii* (reviewed by Kempf 1963; see also Fernández-Marín et al. 2005). The populations at our study sites near Manaus also had very high numbers of nest entrances with a maximum of six nest entrances per square meter in one study plot (Rabeling 2004). Excavations were conducted in areas with a lower density of nest entrances in order to avoid confusion about the nest entrance to which a given chamber belonged. Nonetheless, some chambers were interconnected via lateral tunnels (Figure 1b), so the possibility that distinct *M. smithii* nests may be connected to neighboring nests forming a network of chambers with multiple entrances cannot be excluded.

One way to determine whether an entrance belongs to a single nest, or whether *M. smithii* builds interconnected nest systems, would be to use complementary excavation methods. Williams and Lofgren (1988) were the first to report the use of dental plaster to cast nests of ground dwelling ant species. Orthodontal plaster thinned with water was poured into the nest entrance, the liquid filled the voids of the nest and later the hardened cast could be excavated and reassembled, revealing the three dimensional structure of the nest (see also Tschinkel 2003, 2004). However, this method was problematic if tunnel diameters were small. In *Mycocepurus* the mean tunnel diameter was 2–3 mm and in such a case the liquid dental plaster might harden before it reached the bottom of the nest and several pourings of dental plaster might be required. Alternatively, Tschinkel (2004) used liquid metal, such as zinc or aluminum. He described that the best conditions for nest castings are in sandy soils, where the liquid, either plaster or metal, can displace the air trapped in nest chambers into the surrounding soil. But even though the heavy clay soils of Amazonia would probably provide suboptimal conditions for nest casts and only produce incomplete casts, nest casting and genetic information of the colony's members (see below) should be considered during future excavations in order to yield additional information about the possible connections between *M. smithii* nest systems.

In contrast there is no doubt that all *M. goeldii* colonies in the study area had only one entrance. Other excavations of *M. goeldii* nests near Manaus, Brazil (Mueller personal communication), of *M. obsoletus* from Brazil and of two undescribed *Mycocepurus* species from Manaus, Brazil and Peru (Rabeling in preperation) also revealed a single entrance per colony.

### Nest chambers

The number of chambers per nest varies with the colony's age and is only a snapshot of the colony's life. In the Manaus area, the maximum number of chambers per nest was 15 for *M. smithii* and 21 for *M. goeldii.* In Puerto Rico, nests of reproductively active *M. smithii* colonies had 1–4 and non-reproductive colonies had 2–7 chambers (Fernández-Marín et al. 2005). In both studies, it is possible that not every chamber of a nest system was encountered or that a chamber of a neighboring nest was mistakenly included, especially when nest densities were high.

In both species, chambers with a fungus garden had a larger volume than non-garden chambers. The latter were mostly abandoned chambers and were often partly refilled with soil. The organic content of the chambers seems to attract earthworms, as indicated by the common presence of earthworm feces in these chambers.

### Reproductive biology

Based largely on the absence of males during field studies in Puerto Rico, evidence has accumulated that *M. smithii* is capable of reproducing via thelytokous parthenogenesis (Fernández-Marín 2000, Fernández-Marín et al. 2005). Subsequent laboratory breeding experiments by Himler et al (2004) provide additional support that a Panamanian *M. smithii* population is capable of asexual reproduction. Although it is not known whether parthenogenetic reproduction occurs in the Manaus population, high nest densities and tunnels connecting fungus chambers (of the type shown in Figure 1b) suggest that *M. smithii* could form metacolonies, defined as a set of colonies that are linked through regular exchange of workers and possibly brood, which disperse by a “clonal” growth mechanism. In such metacolonies tunnels connect nest chambers with each other and if the distance from a particular chamber to the original nest entrance becomes too large, workers might establish a secondary entrance. Consequently, the number of nest entrances would not be indicative of the number of individual colonies, and population densities would appear higher on the surface.

The observation that three gynes were found in a single chamber is consistent with the idea that *M. smithii* could disperse through budding. Young gynes might leave their natal chamber and establish a colony in an adjacent chamber, instead of leaving the colony during the nuptial flight as in many ant species. Fernández-Marín et al.'s (2005) hypothesizes that *M. smithii* is secondarily polygynous in Puerto Rico. To further investigate the reproductive biology and geographic distribution of parthenogenesis of *M. smithii* one of us (CR) is currently studying the population biology of *M. smithii* in Latin America using molecular markers.

### Fungus garden organization and development

The lamellate fungus gardens of *M. smithii* and *M. goeldii* were suspended directly from the chamber ceiling. Cultivars of this type have also been observed in *M. smithii* and a new *Mycocepurus* species from Peru (Rabeling unpublished data). Fernández-Marín et al. (2004) suggest that garden placement is correlated with the phylogenetic position of the genus within the attines, based on the phylogenetic hypotheses of Schultz and Meier (1995), Wetterer et al. (1998), Lattke (1999) and Schultz (2000). The most plesiotypic genera, *Mycocepurus*, *Apterostigma* and *Myrmicocrypta*, begin garden cultivation on the detached wings of foundress queens, which are attached to the chamber ceiling. Species in more derived attine genera utilize roots as a platform for fungal growth, including *Trachymyrmex relictus* (Rabeling 2004) and some leafcutter ant species (Weber 1972).

By studying *Mycocepurus* nest systems under field and laboratory conditions, a series of observations have been made on different stages of fungus garden development that suggests a possible sequence for the construction and development of a multi-chamber nests in the genus *Mycocepurus.* We assume that the gyne initially establishes the colony and its fungus garden (for detailed description see Fernández-Marín et al. 2004). Once the first worker generation emerges, they take over all forms of work. During the construction of new chambers workers dig new nest chambers by removing soil until the final chamber size is achieved. Subsequently, fungus cultivation in these new chambers begins by first introducing small pieces of garden, attached to the chamber ceiling. These pieces of mycelium are fed with organic particles from the leaf litter, and grow into mycelial strands hanging down from the top of the chamber (Figure 1d). The final growth stage is reached when the fungus garden fills the entire chamber. The hanging strands of mycelium look like hanging curtains (Figure 1a). The same organization of fungus gardens had been described for *M. smithii* from Puerto Rico (Fernández- Marín et al. 2005). In adult garden chambers one of the colony's gynes (or more in oligogynous colonies) establishes herself in a new garden chamber and begins to lay eggs. The eggs, which are not covered with mycelium as in some attine species, are deposited in small cavities in the mycelium, where they develop into larvae and then pupae.

It is worth noting that even in chambers filled with a fungus garden, the mycelium never touches the chamber walls, an observation also made by Fernández-Marín et al. (2005) for *M. smithii* in Puerto Rico. An excavation of a colony after a heavy rain storm suggested why this might be the case. The walls of the excavated chambers were wet and the water produced a puddle on the chamber floor. Nevertheless, the fungus garden and the brood remained dry. Even if the chamber is very deep, termite, ant and earthworm tunnels drain water into deeper layers (observations made by W. Hanagarth in the same study area), so that the shallower fungus chambers are in danger of flooding. Consequently, the garden chambers are not only maintaining a uniform microclimate for brood development (Townsend 1870), their architecture appears to minimize the risk of flooding and thereby also the outbreak of microbial diseases.

### Colony size

The colony sizes of many plesiotypic attine species are poorly known. Luederwaldt (1918, 1926) described colony sizes of “several hundred” for *M. goeldii.* Weber (1972) gives values for *Myrmicocrypta* and *Apterostigma* colony sizes, which form, together with *Mycocepurus*, the most plesiotypic clade within the Attini. Some *Aptero-stigma* species appear to have small colonies with 17 (*A. tramitis*) to 133 workers (*A. dentigerum*) per colony. A *Myrmicocrypta buenzlii* nest contained 1558 workers (reviewed by Price et al. 2003). The colony sizes of *M. goeldii* and *M. smithii* reported here from Manaus therefore fall within in the range currently known of this group of attines.

Since some workers and gynes inevitably escaped when the chambers were opened, our results represent an underestimate of the actual colony sizes, and some nests, which consequently appear queenless, probably did contain a queen(s). Even though it is not known whether *M. smithii* is polydomous, our population counts for *M. smithii* (10–163) are similar to those made by Fernández-Marín et al. (2005), in which they counted 77.2 ± 41 workers in reproductive nests and 41.6 ± 36.9 workers in non-reproductive nests of *M. smithii* in Puerto Rico.

On average single chambers as well as whole nests of *M. goeldii* were inhabited by significantly more workers than those of *M. smithii.* In addition to having more populous colonies, *M. goeldii* also had larger chambers and larger average worker body size. In both species, one individual was found on average per cubic centimeter of chamber volume.

### Outlook

Detailed data on nests and colony structure of *M. goeldii* and *M. smithii* are necessary for understanding their biology and will help to investigate the hypothesis of asexuality in *M. smithii.* They are also critical for understanding the evolutionary origin of the symbiosis between attine ants, their basidiomycete fungi, and associated microorganisms. The phylogenetic relationships among the most plesiotypic attine genera are currently not well enough resolved to permit a meaningful discussion of what the ancestral nest architecture, fungus-garden organization or fungus cultivar species might have been. Nevertheless, these studies, once they are made possible by future phylogenetic analyses, will be interesting due to the diverse habits of plesiotypic attine species.

The ecological impacts of debris-collecting attines have been largely neglected (see Eschenbrenner 1994). However, *Mycocepurus* ants were observed in high nest densities at our study sites, and by collecting debris and growing fungus gardens in their subterranean chambers, these ants accumulate nutrients in low soil layers, which subsequently become available to higher plants after the chambers are abandoned by the colony. Based on the data presented here on nest density, number of chambers per nest and number of individuals per colony, we are currently working on a reliable statement about the enrichment effect of small fungus-gardening ants on soils, which may be especially important for the geo-chemically impoverished soils of Central Amazonia.
